# Phase II/III multicentre randomised controlled trial evaluating a strategy of primary surgery and adjuvant chemotherapy versus peri-operative chemotherapy for resectable gastric signet ring cell adenocarcinomas – PRODIGE 19 – FFCD1103 – ADCI002

**DOI:** 10.1186/1471-2407-13-281

**Published:** 2013-06-10

**Authors:** Guillaume Piessen, Mathieu Messager, Karine Le Malicot, William B Robb, Frédéric Di Fiore, Marie Guilbert, Marie Moreau, Véronique Christophe, Antoine Adenis, Christophe Mariette

**Affiliations:** 1 Department of Digestive and Oncological Surgery, University Hospital of Lille, F-59037 Lille, France; 2 University Lille Nord de France, F-59037 Lille, France; 3 Inserm, UMR837, Jean-Pierre Aubert Research Center, Team 5 Mucins, Epithelial Differentiation and Carcinogenesis, rue Polonovski, 59045 Lille Cedex, France; 4 Fédération Francophone de Cancérologie Digestive, Medical University, 7 Bd Jeanne d'Arc, BP 87900 F-21079 Dijon cedex, France; 5 Department of Gastroenterology, Digestive Oncology Unit, Rouen University Hospital, 1 rue de Germont, F-76031 Rouen Cedex, France; 6 University Lille Nord de France - Université de Lille-III, URECA EA 1059, BP 60149 F-59653 Villeneuve-d'Ascq Cedex, France; 7 Gastrointestinal Oncology Department, CLCC Oscar Lambret Comprehensive Cancer Center, 3 rue Combemale, F-59020 Lille cedex, France

**Keywords:** Signet ring cells, Gastric adenocarcinoma, Surgery, Perioperative chemotherapy, Randomized study, Emotion, Couple

## Abstract

**Background:**

A dramatic increase in the incidence of the diffuse form of gastric adenocarcinomas and particularly signet ring cell carcinomas has been observed in Western countries. Evidence is accruing that signet ring cell carcinomas may have inherent chemo resistance leaving many clinicians unsure of the benefits of delaying surgery to pursue a neoadjuvant approach.

**Methods/design:**

PRODIGE-19-FFCD1103-ADCI002 is a prospective multicentre controlled randomised phase II/III trial comparing current standard of care of perioperative chemotherapy (2x3 cycles of Epirubicin, cisplatin, 5-fluorouracil) with a strategy of primary surgery followed by adjuvant chemotherapy (6 cycles of Epirubicin, cisplatin, 5-fluorouracil) in patients with a stage IB-III gastric signet ring cell tumour. The principal objective of the phase II study (84 patients) is to determine if the experimental arm (primary surgery followed by adjuvant chemotherapy) has sufficient interest in terms of percentage of living patients at 24 months to be evaluated in a phase III trial. If 7 or less patients in the experimental arm are alive at 24 months, phase III will not be initiated. The primary objective of phase III (230 additional patients) is to demonstrate superiority of the experimental arm in terms of overall survival. Secondary endpoints include overall survival at 36 months, disease free survival at 24 and 36 months, R0 resection rates, treatment tolerance, postoperative mortality and morbidity evaluated by Clavien-Dindo severity index, the prognostic impact of positive peritoneal cytology and the assessment of quality of life. An ancillary study will assess the emotional and cognitive impact of surgery and perioperative chemotherapy for both the patient and their partner.

**Discussion:**

As inherent chemo resistance of signet ring cell tumours and delay in definitive surgery may favour tumour progression we hypothesise that a policy of primary surgery followed by adjuvant chemotherapy will improve overall survival compared to a standard perioperative chemotherapeutic strategy. This randomised phase II/III trial is the first dedicated to this histological subtype. Whilst the development of new biomarkers and targeted therapies are awaited, the results of this trial should further help in devising individualised protocols of patient care in a tumour group whose diversity increasingly demands assessment of alternative strategies.

**Trial registration:**

ClinicalTrials.gov, NCT01717924

## Background

Gastric adenocarcinoma (GA), despite its decreasing overall incidence, still remains the 5th most frequently diagnosed cancer and the second most common cause of cancer related death worldwide [1,2]. It is a heterogeneous disease with two histological classifications mainly being used. The Lauren classification [3] primarily differentiates between “intestinal” and “diffuse” types. The intestinal type corresponds to tubular or villous well-differentiated GA whereas the diffuse type corresponds to less differentiated and more infiltrative GA and includes signet ring cell adenocarcinomas (SRCs). As GA is typically a mixture of histological patterns, the World Health Organisation classification [4] has defined SRCs as an adenocarcinoma in which the predominant component (more than 50% of the tumour) consists of isolated or small groups of malignant cells containing intracytoplasmic mucins. A dramatic increase in the incidence of the diffuse form of gastric cancer and particularly SRCs has been observed, mainly in Western [5,6]. Consequently, in the most recent western studies, SRCs represents 16% to 45.4% of GAs [5,7,8].

The phase III trials [9,10] which have established peri-operative chemotherapy as the European standard of treatment for gastric cancers provide no subgroup analysis of the efficacy of this strategy for SRC tumours. A few retrospective [11-13] and prospective publications [14], with limited numbers of patients, have however suggested SRCs may have inherent chemo resistance. These observations have left many clinicians unsure of the benefits of delaying definitive surgery and pursuing a neoadjuvant approach for a tumour which behaves aggressively and displays significant resistance to current chemotherapy.

To support these observations and establish more reliable data regarding the behaviour of SRC tumours, at the start of 2010 our team launched a French retrospective multicenter study of all consecutive oesophagogastric adenocarcinomas in 19 centres in France treated between January 1997 and January 2010 (ADCI001) [8]. Amongst 3010 patients registered 1050 (34.9%) had a SRC tumour, with 126 being metastatic at diagnosis and excluded from the study. The remaining 924 patients underwent an intention to treat analysis according to whether they underwent primary surgery (n = 753, 81.5%) or neoadjuvant chemotherapy (n = 171, 18.5%). Multivariate analysis showed pre-operative chemotherapy to be an independent predictor of poor survival (HR = 1.4, 95% CI 1.1-1.9, p = 0.042), with overall survival at 2 years of 27.1% in the primary surgery group versus 12.3% in patients treated with neoadjuvant chemotherapy. Hence, increasingly data is suggestive of SRC chemo resistance, and a deleterious effect of delaying definitive surgery for an ineffective treatment whose toxicity may cause host immunosuppression facilitating disease progression.

There is therefore clear impetus for a prospective randomised study to evaluate the appropriate perioperative therapeutic strategy for SRCs.

## Methods/design

### Protocol overview

PRODIGE-19-FFCD1103-ADCI002 is a prospective multicentre controlled randomised phase II/III trial comparing current standard of care (2x3 cycles of perioperative Epirubicin, cisplatin, 5-fluorouracil (ECF) chemotherapy) with a strategy of primary surgery followed by adjuvant ECF chemotherapy (6 cycles) in patients with a SRC tumour and eligible for curative surgery. In phase II patients will be randomised using minimisation with stratification criteria – centre, tumour stage, performance status and tumour localisation. The principal objective will be to determine if the experimental arm (primary surgery followed by adjuvant chemotherapy) has a sufficient interest in terms of percentage of living patients at 24 months to be evaluated in a phase III trial. If ≤10% of patients in the experimental arm is alive at 24 months, phase III will not be initiated. Recruitment will be postponed for a 6 month period after the completion of phase II of the study to allow for the interim analysis of data. The primary objective of phase III will be to demonstrate the superiority of the experimental arm in terms of overall survival. The study is planned for a total duration of 10 years and is registered on clinicaltrial.gov website (NCT01717924).

### Inclusion criteria

PRODIGE-19 will include patients with an adenocarcinoma of the stomach and Siewert type III tumours of the oesophago-gastric junction, with histological proof on pre-therapeutic biopsies of either a SRC tumour or diffuse type tumour according to the Lauren classification. Reliability of samples obtained from routine pre-treatment endoscopic biopsies to predict SRC histology has been recently demonstrated by our group [15].

All tumours of stage IB to III will be included (T1N+, T2N0 N+, T3N0 N+, T4N0 N+) as staged by routine endoscopic ultrasound examination (EUS), or by computerised tomography if the tumour is non-traversable at EUS. Patients will be between the ages of 18 and 80, must be assessed as suitable for curative surgery with the absence of visceral metastases or evidence of peritoneal carcinomatosis during a mandatory pretherapeutic staging laparoscopy. All included patients must have a weight loss of less ≤15% of body mass, have a World Health Organisation performance status of ≤2, with an ejection fraction of ≥50% prior to the administration of Epirubicin and must not have been previously treated with either chemotherapy or radiotherapy for a gastric cancer. Patients must have a neutrophil count of ≥1500/mm^3^, platelet count of ≥100,000/mm^3^, a creatinine clearance of >50ml/minute and a total Bilirubin <1.5 times the upper limit of normal. All patients must give their informed consent for participation in this trial.

### Exclusion criteria

All patients who do not meet the inclusion criteria will be excluded. Other contraindications to participation include tumours classified as T1N0 by preoperative staging, the presence of other malignant tumours treated with curative intent within 5 years (with the exception of basal cell carcinoma of the skin and carcinoma in situ of the cervix), patients previously treated with abdominal or thoracic radiation and patients with a known allergy to one of the trial medications. Current pregnancy or breastfeeding will render females ineligible. Evolving cardiac, renal and respiratory insufficiency, as well as myocardial infarction within 6 months and known Child’s B or C cirrhosis will all also warrant patient exclusion. Finally patients who are not capable for family, social, psychological or geographical reasons to attend for regular follow-up and who do not have legal capacity will not be included.

### Endpoints of trial

The primary endpoint of this trial will be the evaluation of the percentage of patients still alive 24 months after randomisation. The secondary endpoints for this trial include, overall survival (OS) at 36 months, disease free survival (DFS) at 24 and 36 months, R0 resection rates, treatment tolerance, postoperative mortality and morbidity evaluated by Clavien-Dindo severity index [16], rates of completion of all elements of the planned therapy, the prognostic impact of positive peritoneal cytology and the assessment of quality of life (EORTC QLC-C30 and EORTC STO 22). A complementary study, subsequently described, will assess the emotional and cognitive impact of surgery and perioperative chemotherapy for both the patient and their partner.

OS is defined as the time interval between inclusion and death from whatever cause and patients lost to follow-up will be censured at the last date of follow-up. Disease free survival will be defined as the time interval between the date of inclusion and the date of first recurrence or the date of death in the absence of disease recurrence. The R0 resection rate will correspond to the percentage of patients benefitting from a micro and macroscopically complete resection [17]. Neoadjuvant and/or adjuvant treatment tolerance will be estimated according to the National Cancer Institute Toxicity Criteria (NCI-CTC version 4.0). Evaluation of the uptake of all elements of planned treatment will be performed and quality of life questionnaires and human and social sciences questionnaires will be completed at 4 defined time-points – randomisation, the eve of commencement of treatment, 1 month (+/−7 days) after leaving hospital following surgery and 1 month (+/−7 days) after completing adjuvant chemotherapy. The quality of life questionnaires will also be completed at each 4 monthly follow-up visit.

### Randomisation

Randomisation will be performed by minimisation with stratification criteria – centre, tumour stage, performance status and tumour localisation. The maximum delay between randomisation and either the start of neoadjuvant treatment or surgery will be 4 weeks. A randomisation number and the treatment allocation are sent by facsimile communication by the “Centre de Randomisation-Gestion-Analyse” (CRGA) at the Fédération Francophone de Cancérologie Digestive (FFCD) headquarters in Dijon, France to the investigator after the receipt of the inclusion form.

### Pre-therapeutic work-up

Prior to inclusion in the trial an information booklet will be given to each patient and the trial protocol will be clearly explained. The initial work-up must take place in the 4 weeks preceding randomisation and will comprise of the following:

i) A complete physical examination.

ii) Estimation of full blood count, calculation of creatinine clearance using the Cockcroft formula, measurements of electrolytes, total protein, albumin, alkaline phosphatase, aspartate transaminase, alkaline transaminase, total and conjugated Bilirubin, gamma-GT, pregnancy test for females of reproductive years and discussion of contraceptive methods during the trial period.

iii) Tumour markers evaluation – carcinoembryonic antigen and carbohydrate antigen 19.9.

iv) Assessment of operability – electrocardiogram, cardiac echography, anaesthetic consultation and posterior-anterior and lateral chest x-ray (not necessary where a CT thorax has been performed).

v) Oesophago-gastro-duodenoscopy with multiple biopsies and examination by retroflexion.

vi) Contrast study.

vii) Contrast enhanced computerised tomography of the thorax, abdomen and pelvis.

viii) Endoscopic ultrasound will be performed systematically. The pre-therapeutic TNM classification will be made by EUS, except for tumours which cannot be traversed by EUS, in which case the TNM stage will be based on CT findings.

ix) Staging laparoscopy will be performed systematically. All suspicious lesions will be biopsied and a laparoscopic feeding jejunostomy will be performed for patients who have lost >10% of habitual body mass. Peritoneal fluid, if already present, will be sampled prior to any manipulation of the tumour and if the sample gives <50 mls, 100mls of normal saline will be instilled around the primary tumour and 50mls recuperated from the epigastric/left hypochondriac regions after a delay of 2 minutes without tumour manipulation. In cases of the discovery of peritoneal carcinomatosis, peritoneal cytology will not be performed.

x) Positron emission tomography scanning will not be routinely performed due to the known weak tracer uptake specific to SRC tumours [14].

### Treatment methods

**Figure 1:**
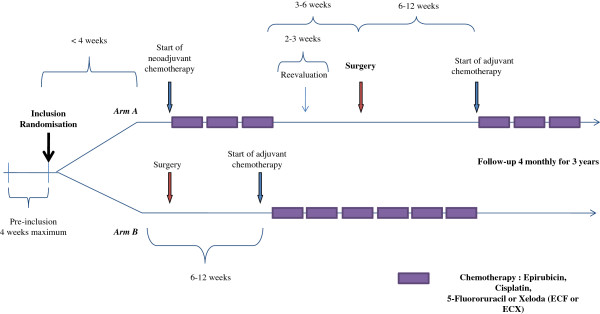
Therapeutic scheme for the protocol.

The therapeutic scheme for this trial is depicted in Figure 1.

### Chemotherapy regimens

#### Peri-operative chemotherapy (group A)

Patients randomised to group A will receive the established European standard of treatment– 3 pre- and postoperative cycles of ECF. Neoadjuvant treatment will commence within 4 weeks of randomisation and each cycle will last 21 days. Epirubicin will be administered at a dose of 50 mg/m^2^ by intravenous infusion over 30 minutes on day 1 followed by intravenous infusion over 60 minutes of cisplatin at a dose of 60 mg/m^2^ and then continuous infusion of 5-fluorouracil from days 1–21 at a dose of 200 mg/m^2^/day. It will be permitted to replace infusion of 5-fluorouracil with its oral equivalent (capecitabine) depending on the normal treatment habits of each centre.

Patients will undergo clinical and staging re-evaluation 2–3 weeks after completion of the neoadjuvant regimen and surgical resection will be performed 3–6 weeks after the completion of neoadjuvant chemotherapy. Adjuvant chemotherapy will commence 6–12 weeks after surgery and will comprise the same regimen where there has not been disease progression or severe toxicity during the neoadjuvant phase. If either of these events has occurred the administration of the adjuvant chemotherapy or the protocol for its substitution will be left to the decision of the local multi-disciplinary team meeting. Clinical, haematological and toxicity evaluations will be carried out prior to administration of each cycle of chemotherapy and an echocardiogram will be performed prior to neoadjuvant and adjuvant treatments and at the conclusion of adjuvant treatment.

#### Adjuvant chemotherapy regimen - group B

Patients randomised to group B will undergo primary surgery followed by 6 cycles of adjuvant ECF chemotherapy. Primary surgical resection will take place within 4 weeks of randomisation. Six cycles of adjuvant chemotherapy will commence 6–12 weeks after surgical resection. Each cycle of chemotherapy will consist of the same regimen of ECF as used for group A and at the same dosage.

### Re-staging post neoadjuvant chemotherapy (group A)

Tumoural restaging will be performed 2–3 weeks after the end of neoadjuvant chemotherapy. It will comprise of the following examinations; clinical examination, same blood analyses as at inclusion [see *Pre*-*Therapeutic Work*-*Up* (*ii*)], tumour marker estimation (CEA and CA19.9), anaesthetic consultation, oesophago-gastro-duodenoscopy with multiple biopsies and retrovision, radiological contrast study, CT thorax, abdomen and pelvis and EUS evaluation. This re-evaluation will be performed by the same team and by the same technique as the initial work-up.

### Surgical Resection

All surgical resections will be performed by open laparotomy. At the start of definitive surgical resection, peritoneal cytology will be performed in order to study the effects of, (a) preoperative chemotherapy on the results of cytology performed during staging laparoscopy in group A and, (b) the effect on peritoneal cytology of the interval between exploratory laparoscopy and definitive resection in group B.

Surgical resection will follow the French national guidelines for GA, published by the SFCD-ACHBT-HAS-INCA [18]. In view of the infiltrative nature of SRC tumours, a total gastrectomy is recommended irrespective of the gastric tumour location. Nevertheless, for small distal tumours, a partial gastrectomy may be considered on a case by case basis whilst safeguarding the usual oncological principles of resection. For Siewert type III tumours of the gastro-oesophageal junction the recommendations for resection will apply apart from the requirement of an 8 cm proximal tumour free resection margin. When resection is extended to the lower oesophagus, either transhiatally or via thoracotomy, the lower oesophagus will be resected en bloc with a lower mediastinal lymphadenectomy. Frozen section analysis of the proximal and distal resection margins will be routinely performed. An enlarged resection to adjacent organs will be performed when necessary to obtain an R0 resection and, when noted, resection of peri-tumoural localised carcinomatosis will also be performed. A D2 lymphadenectomy without distal pancreatectomy and splenectomy will be standard, with the harvest of at least 25 lymph nodes. In both treatment arms the trial protocol recommends the placement of a feeding jejunostomy to limit post-operative weight loss and to help facilitate tolerance of subsequent adjuvant therapy.

### Standards of peri-operative care

Patients will be required to abstain from smoking and alcohol for 1 month pre-operatively. Supplemental enteral nutrition will be given to patients who have lost >10% of their baseline body mass and all patients will receive 7 days of pre-operative immunonutrition by Oral Impact® independent of their nutritional status [19].

All patients will receive appropriate antibiotic prophylaxis at induction of anaesthesia and post-operative analgesia will be by patient controlled epidural analgesia (PCEA) or alternatively by standard patient controlled analgesia (PCA). Post-operative nutrition will be commenced on day 2 or 3 post-operation via the jejunostomy and at an initial low volume (250 mls) and increased incrementally. Deep vein prophylaxis will be administered routinely by low molecular weight heparin. Chest physiotherapy and post-operative incentive spirometry will be standard for all patients. In cases where an anastomotic leak is suspected the investigation of reference will be an upper gastro-intestinal endoscopy, or alternatively a CT scan with both injection and ingestion of contrast medium.

### Histological analysis

Pathological examination of the operative specimen must report upon: tumour location, type of operation, details of lymphadenectomy and detailed description or lymph node stations, a macroscopic tumour description, definitive histological classification, documentation of the % of signet ring type cells, Lauren classification, definitive results of per-operative frozen sections, pTNM/ypTNM stage according to the 2009 7th edition of the UICC classification for gastric cancers, resection radicality (R0, R1, R2) and documentation of histological tumour response after neoadjuvant chemotherapy.

### Patient follow-Up after surgery

Participating patients will be reviewed in out-patients 1 month (+/−7 days) after hospital discharge. They will have a full clinical examination. Questionnaires will be completed with regards to quality of life (QoL), emotional state, emotional adjustment, perception of illness and lifestyle burden. A further consultation will be scheduled for one month (+/−7days) after completion of adjuvant chemotherapy with evaluation of clinical state, treatment tolerance, haematological parameters, renal function, tumour markers, evaluation of cardiac ejection fraction after Epirubicin by echocardiogram and the same set of questionnaires will be completed.

All participants will subsequently be followed for 3 years after randomisation. Out-patient consultations will be scheduled every 4 months (+/−15 days) and patients will be evaluated by clinical examination, thoraco-abdominal CT scan, tumour marker estimation (CEA and CA19.9) and QoL questionnaires. Further examinations will be requested as required. If disease recurrence is suspected its site (loco regional or distant) must be documented by the appropriate investigations and histological proof be obtained wherever possible. In cases of strong clinical suspicion of recurrence without radiological or histological proof, surgical exploration is recommended to identify peritoneal carcinomatosis not visualised by standard radiological investigation. All cases of disease recurrence will be discussed at the multi-disciplinary team meeting and further therapeutic strategies recorded.

### Reasons for discontinuing treatment protocol

The treatment protocol may be discontinued for the following reasons: decision of the treating doctor, high grade toxicity, occurrence of a serious adverse effect, disease progression, patient refusal to continue and patient death. In cases where treatment is discontinued, patients will remain within the intention to treat analysis.

### Statistical evaluation and sample size calculation

Phase II– The phase II part of this trial will determine the percentage of patients in the experimental limb of the study (group B) who are alive at 24 months and thus validate the interest pursuing the evaluation of a strategy of primary surgery in a subsequent phase III trial. To demonstrate the interest of the experimental arm of this study, it will be necessary to include 40 in each treatment arm, with a unilateral alpha error of 5% and a power of 85%. Taking into account a 5% level of loss to follow-up, 84 patients will be included in total (42 in each treatment arm). If 7 or less patients are alive after 24 months, then the treatment B will be considered not interesting. Survival data for patients from phase II of this trial will allow for the readjustment of the number of patients required to be enrolled in the phase III trial according to the adaptive trial design.

Phase III – The objective of the phase III trial will be to demonstrate the superiority of group B and the principal outcome criteria will be overall survival. To demonstrate a desired 10% difference in overall survival in favour of group B at 24 months, with a bilateral alpha error of 5% and a power of 85%, it will be necessary to observe 291 deaths. With an estimated rate of inclusion of 6 patients per month, with a 5% rate of loss to follow-up and surveillance of the last included patient of 24 months, a total of 314 patients will have to be recruited. On this basis 230 additional patients will need to be recruited after the completion of phase II.

Two intermediate analyses are planned, one for Phase III sample size read justement and the second one to allow for the early identification of the superiority of group B. The first intermediate analysis of survival data will take place at the end of phase II. The second intermediate analysis will be performed after either 175 deaths or 60% of events have occurred.

Statistical analysis for phase II will be performed on a modified intention to treat population meaning all patients who can be evaluated and who are not lost to follow up at 24 months. For phase III, analysis of the main criteria will be performed on an intention to treat (ITT) principle. The per-protocol population will be defined as the ITT population having no major deviation or major of the protocol.

Continuous variables will be described by their mean and standard deviation or median, Interquartile Interval (Q1-Q3) and range. Survival endpoints will be estimated using the Kaplan Meier method, described by median values with 95% confidence Interval and compared in Phase III using the log-rank test. QoL data will be analysed according to treatment arm and treatment tolerance assessed using the National Cancer Institute Toxicity Criteria – (NCI CTC). The Clavien-Dindo scale will be used to assess peri-operative complications [16]. Cox univariate and multivariate analyses will allow exploratory analyses of prognostic factors, time until deterioration of QoL and disease free survival.

### Scheme for trial analysis (Figure 2)

#### Ancillary study

An ancillary study of Human and Social Sciences will be performed entitled “Emotional and Cognitive Impact of Surgery and Peri-Operative Chemotherapy on Patients and Partners Diagnosed With a Gastric Adenocarcinoma”. The principal aim of this novel exploratory study is the longitudinal assessment of the emotional and cognitive state of the patient and partner according to allocated treatment arm. Evaluation will be by the following list of questionnaires (Quality of Life - QLQ-C30 and QLQ-STO-22 for the patient and SF-36 for partner, Emotional State – depression - CES-D and anxiety – STAI-Y-A for patient and partner, Emotional Adjustment – WCC for patient and partner, Perception of Disease – Brief-IPQ for patient and partner, Comprehension of Trial – ICEC-R for patient and partner, and Burden Assessment – CRA for patient and partner). Assessment will take place at 4 measured time-points – randomisation, day prior to surgery, prior to adjuvant treatment and on stopping adjuvant treatment. This study will allow to know better which therapeutic strategy, among the 3 compared, is the best for the patient’s and their partner’s quality of life and emotional state, which is determining in therapeutic decisions and compliance to treatments, what coping strategies promote the patient’s and their partner’s quality of life and emotional state the best. This result will be particularly interesting to improve the psycho-oncological care of the patient-partner couple: what perceptions the patient and their partner have about the disease and clinical trials, which are determining factors in therapeutic compliance, and that need to be known to improve psycho-oncological support in particular.

**Figure 2:**
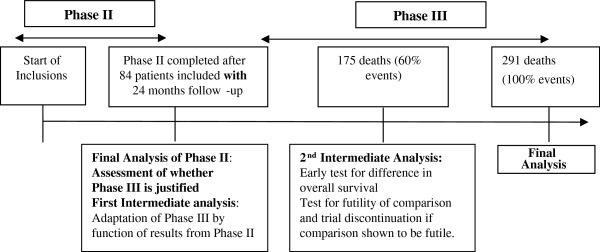
Scheme for trial analysis.

#### Ethical considerations

The study protocol was approved by the Institutional Review Board, the Nord Ouest IV ethics committee on the 15 of May 2012 and the Agence Nationale de sécurité des médicaments et des produits de santé (ANSM) on the 26 of June 2012 under the number 2012-000998-24. The institutional promoter is the University Hospital of Lille, France. The trial has been registered on the ClinicalTrial.gov website under the identification number NCT01717924. This study has received grant funding from the French National Cancer Institute (INCA) in 2011. The study complies with the Declaration of Helsinki rules and the principles of Good Clinical Practice guidelines. Informed consent will be obtained from each patient in written form prior to randomisation and an information leaflet will be given to each patient prior to inclusion in the study. Patient safety and all potential threats to patient safety will be monitored by an independent monitoring board which will be able, taking into account undesirable events and the level of post-operative mortality, recommend the discontinuation of one of the treatments evaluated.

## Discussion

Despite considerable advances in treatment, gastric cancer remains a disease with a poor prognosis. The heterogeneity of this disease, the distinct histological subtypes and the intrinsic molecular diversity represent a challenge and an opportunity in developing individually tailored therapy. The incidence of diffuse type GA, particularly SRC type has been increasing and SRCs represents 16% to 45.4% of GA in recent Western studies [5,7,8]. Tumours of SRC histology have several characteristics which differentiate them from other tumour types. They appear to affect patients at an earlier age [12,20], typically present with a more advanced tumour stage at diagnosis and carry a worse prognosis [12].

The advantage of perioperative treatment strategies for gastric cancer has been demonstrated in several phase III randomised controlled trials. The MAGIC study [9] conducted in the United Kingdom tested the potential efficacy, in 503 patients (stage ≥ II), of 3 cycles of neoadjuvant triplet chemotherapy using cisplatin, 5-fluorouracil and Epirubicin followed by surgery and three cycles of adjuvant chemotherapy compared with surgery alone for adenocarcinomas of the lower œsophagus and stomach. The results are well known, with a significant down staging in the chemotherapy arm in terms of size of tumour (p < 0.001) and a significant improvement in overall survival after 5 years (36% perioperative chemotherapy vs. 23% surgery alone, p = 0.009). This perioperative treatment regimen remains the standard of treatment in Europe. The FNCLCC ACCORD 07-FFCD 9703 trial [10] included 224 patients with an adenocarcinoma of the lower œsophagus and oesophago-gastric junction. Surgery alone was compared to perioperative chemotherapy with cisplatin and 5-fluorouracil, with results essentially confirming the findings of the MAGIC trial. The R0 resection rates were 87% and 74% respectively (p = 0.04) and the overall survival rate at 5 years was 38% vs. 24%, respectively, (p = 0.021). Neither of these trials performed a sub-group analysis for patients with a SRC. Further, preliminary data has suggested the need for a specific treatment strategy for SRCs because of a more advanced stage at diagnosis, with a greater prevalence of lymph node invasion and peritoneal carcinomatosis, a lower rate of R0 resection, earlier and more frequent disease recurrence and finally a worse overall prognosis [12].

Recently Messager et al [8] have published the results of a large multicentre comparative cohort study investigating the impact of peri-operative chemotherapy on survival in patients with SRC gastric tumours. It represents the largest study conducted on this histological subtype and involved analysis of 1050 patients diagnosed with SRC gastric tumours. No survival benefit was demonstrated for patients with SRC histology receiving peri-operative chemotherapy. Nor did neoadjuvant therapy result in tumour down-staging, down-staging of nodal disease, or decrease the disease recurrence. Indeed the authors found some evidence that disease progression during neoadjuvant treatment may have occurred in this subgroup of patients - extended resections were more commonly required and a longer median survival for patients treated with surgery alone prior to the incorporation of peri-oerative chemotherapy into the French guidelines for GA in 2006. This provides a clear imperative of the assessment of an alternative treatment strategy.

As several studies [11-14] have suggested a relative chemo resistance for SRC tumours and delay in definitive surgery may favour tumour progression [8] we hypothesise that a policy of primary surgery for SRC tumours, followed by adjuvant chemotherapy, will improve overall survival at 2 years when compared to a standard peri-operative chemotherapeutic strategy. This primary outcome criteria is justified as it provides a short term evaluation criteria (important in view of the very poor prognosis associated with this disease) and survival at 24 months has been found in a multicentre retrospective series of over 1000 patients to be significantly different (27% vs. 10%) [8].

This randomised trial will provide high level evidence regarding the value of a strategy of primary surgery for SRC gastric tumours. Whilst the development of new biomarkers and associated targeted therapies are awaited to individualise gastric cancer therapy, the results of this trial should will help further in devising individualised protocols of patient care in a tumour group whose diversity increasingly demands assessment of alternative strategies.

## Abbreviations

GA: Gastric adenocarcinoma; SRC: Signet ring cell; ECF: Epirubicin, cisplatin, 5-fluorouracil; EUS: Endoscopic ultrasound; OS: Overall survival; DFS: Disease free survival; FFCD: Fédération Francophone de Chirurgie Digestive; CEA: Carcinoembryonic antigen; CA: Carbohydrate antigen; PCEA: Patient controlled epidural analgesia; PCA: Patient controlled analgesia; CT: Computerised tomography; QoL: Quality of life; EORTC: European organisation for research and treatment of cancer; 95% CI: 95% confidence interval; WHO: World Health Organisation; cTNM: Clinical tumoural staging; NCI CTC: National Cancer Institute Clinical Toxicity Criteria; ANSM: Agence Nationale de sécurité des médicaments et des produits de santé; SFCD-ACHBT-HAS-INCA: Société Française de Chirurgie Digestive – Association Française de Chirurgie Hépato-Biliaire et de Transplantation Hépatique - Haute Autorité de Santé – Institut National de Cancer.

## Competing interests

The authors declare that they have no competing interests.

## Authors’ contributions

WBR, GP and CM have been involved in drafting the manuscript; KLM was the statistical advisor; GP, MM, KLM, WBR, FDF, MG, MM, VC, AA and CM have been involved in the study conception and design, assisted in writing the manuscript and have given final approval of the version to be published. All authors read and approved the final manuscript.

## Author information

CM is the study coordinator, obtained the grant and is responsible for the present paper, AA is the co-coordinator of the study, and VC is responsible for the ancillary study.

## Pre-publication history

The pre-publication history for this paper can be accessed here:

http://www.biomedcentral.com/1471-2407/13/281/prepub
